# Low-Dose Mixture Hypothesis of Carcinogenesis Workshop: Scientific Underpinnings and Research Recommendations

**DOI:** 10.1289/EHP411

**Published:** 2016-08-12

**Authors:** Mark F. Miller, William H. Goodson, Masoud H. Manjili, Nicole Kleinstreuer, William H. Bisson, Leroy Lowe

**Affiliations:** 1 National Institute of Environmental Health Sciences, National Institutes of Health, Department of Health and Human Services, Research Triangle Park, North Carolina, USA; 2 California Pacific Medical Center Research Institute, San Francisco, California, USA; 3 Department of Microbiology and Immunology, Virginia Commonwealth University, Massey Cancer Center, Richmond, Virginia, USA; 4 Environmental and Molecular Toxicology, Oregon State University, Corvallis, Oregon, USA; 5 Getting to Know Cancer, Truro, Nova Scotia, Canada; 6 Lancaster Environment Centre, Lancaster University, Bailrigg, Lancaster, United Kingdom

## Abstract

**Background:**

The current single-chemical-as-carcinogen risk assessment paradigm might underestimate or miss the cumulative effects of exposure to chemical mixtures, as highlighted in recent work from the Halifax Project. This is particularly important for chemical exposures in the low-dose range that may be affecting crucial cancer hallmark mechanisms that serve to enable carcinogenesis.

**Objective:**

Could ongoing low-dose exposures to a mixture of commonly encountered environmental chemicals produce effects in concert that lead to carcinogenesis? A workshop held at the NIEHS in August 2015 evaluated the scientific support for the low-dose mixture hypothesis of carcinogenesis and developed a research agenda. Here we describe the science that supports this novel theory, identify knowledge gaps, recommend future methodologies, and explore preventative risk assessment and policy decision-making that incorporates cancer biology, environmental health science, translational toxicology, and clinical epidemiology.

**Discussion:**

and Conclusions: The theoretical merits of the low-dose carcinogenesis hypothesis are well founded with clear biological relevance, and therefore, the premise warrants further investigation. Expert recommendations include the need for better insights into the ways in which noncarcinogenic constituents might combine to uniquely affect the process of cellular transformation (in vitro) and environmental carcinogenesis (in vivo), including investigations of the role of key defense mechanisms in maintaining transformed cells in a dormant state. The scientific community will need to acknowledge limitations of animal-based models in predicting human responses; evaluate biological events leading to carcinogenesis both spatially and temporally; examine the overlap between measurable cancer hallmarks and characteristics of carcinogens; incorporate epigenetic biomarkers, in silico modelling, high-performance computing and high-resolution imaging, microbiome, metabolomics, and transcriptomics into future research efforts; and build molecular annotations of network perturbations. The restructuring of many existing regulatory frameworks will require adequate testing of relevant environmental mixtures to build a critical mass of evidence on which to base policy decisions.

**Citation:**

Miller MF, Goodson WH III, Manjili MH, Kleinstreuer N, Bisson WH, Lowe L. 2017. Low-Dose Mixture Hypothesis of Carcinogenesis Workshop: scientific underpinnings and research recommendations. Environ Health Perspect 125:163–169; http://dx.doi.org/10.1289/EHP411

## Introduction

In August 2015, the National Institute of Environmental Health Sciences (NIEHS) sponsored a workshop that evaluated the scientific support for the low-dose mixture hypothesis of carcinogenesis and developed a research agenda to identify and address critical information gaps. The primary question being addressed was whether ongoing low-dose exposures to a mixture of seemingly safe environmental chemicals produce effects in concert that lead to carcinogenesis, even though the individual chemicals are not classified as carcinogens. The workshop brought together more than 75 participants who engaged in extensive discussions to *a*) explore and identify the most critical information gaps that need to be investigated to better inform the low-dose mixture hypothesis and provide recommendations for future research; *b*) explore and identify key methodologies that will provide enhanced hypothesis testing approaches from a research standpoint; and *c*) identify opportunities to leverage the new information generated by low-dose mixture testing to better inform protective and preventative risk assessment and policy decision-making.

A key aspect of this meeting was the engagement of participants from a wide variety of medical and scientific disciplines, including clinicians, cancer biologists, toxicologists, risk assessors, and epidemiologists from government, industry, and nongovernmental organizations (NGOs). The low-dose mixture hypothesis of carcinogenesis is innately an environmental and public health issue that requires broad expertise to fully consider. Typically, these researchers do not work closely but were brought together to provide a complete systems approach to the hypothesis assessment and the development of future directions.

The hallmarks of cancer were initially summarized by Hanahan and Weinberg (2000) to define the biological processes through which cancer may develop. The hallmarks include the tumor microenvironment, genetic instability, tumor-promoting inflammation, sustained growth signalling, evading anti-growth signalling, replicative immortality, resistance to apoptosis, dysregulated cellular metabolism, immune-system evasion, angiogenesis, and tissue invasion and metastasis. The biology associated with the hallmark characteristics (e.g., systemic and cellular dysfunctions) play a large role in determining whether a specific disruption, or combination of disruptions, will result in tumorigenesis. In 2012, participants at two workshops convened by the International Agency for Research on Cancer (IARC) in Lyon, France, concluded the human carcinogens (Group 1) frequently exhibit 1 or more of 10 key characteristics. A recent paper described these 10 characteristics as an intrinsic property of each individual human carcinogen to induce and encompass multiple mechanistic end points (Smith et al. 2016). Further, a literature-based review evaluating the evidence related to individual chemicals inducing some but not necessarily all of the hallmarks of cancer as defined by Hanahan and Weinberg (2011) has been published by an international task force of 174 scientists from 26 countries in the Halifax Project. This work was released in June 2015 as a special issue of *Carcinogenesis*, and it contains a capstone article (Goodson et al. 2015) and 11 reviews (Nahta et al. 2015; Carnero et al. 2015; Engström et al. 2015; Langie et al. 2015; Narayanan et al. 2015; Kravchenko et al. 2015; Ochieng et al. 2015; Casey et al. 2015; Hu et al. 2015; Robey et al. 2015; Thompson et al. 2015).

The underlying concept of this work posits that if individual chemicals can induce some but not all of the hallmarks of cancer, then combinations of chemicals at low doses may be able to act through different modes of action in concert to induce carcinogenesis. In total, the Halifax Project reviewed 85 chemicals with hallmark-inducing actions on key pathways and mechanisms related to carcinogenesis, with 15% found to have evidence of a dose–response threshold, 59% with evidence of effects in a low-dose range, and no dose–response information was found for the remaining 26%. In essence, the Halifax papers highlighted longstanding concerns over the number of chemical exposures that our populations face. Further, the papers demonstrate that there are a significant number of ubiquitous environmental contaminants that exert nongenotoxic, low-dose effects through hallmark-enabling mechanisms and believed to be instrumental in carcinogenesis. This appears to substantiate the possibility that low-dose exposures to mixtures of these and other chemicals may be contributing to environmental carcinogenesis. For example, a given chemical might be supporting carcinogenesis even though it is not in and of itself a complete carcinogen. One chemical might support two hallmarks, another chemical a third hallmark, and so forth until the sum of results is the same as though there had been an exposure to a single complete carcinogen.

Low-dose exposures have previously been defined by the National Toxicology Program (NTP) as those occurring within the range of typical human exposures (Melnick 2002). It has been well established that hormones act at low concentrations, and as such, the endocrine disruption literature dominates discussions of low-dose effects. Similarly, mixture effects have been well established in the environmental sciences with a focus on cancer (e.g., van den Berg et al. 1998) and non-cancer end points (e.g., Howdeshell 2008). Chemical mixtures may affect biological systems through dose-additive or effect-additive models, or both. The current low-dose mixture hypothesis of carcinogenesis primarily employs an effect-additive model, whereby each individual chemical within a mixture has distinct effects on a given hallmark with the cumulative outcome of those individual impacts being carcinogenesis. Although the effect-additive model was identified at the NIEHS workshop as the primary model for this hypothesis, an additional concern is the possibility of dose-cumulative effects within a given hallmark by two or more chemicals.

From a risk assessment standpoint, this comprehensive review of the biology of cancer also illuminated several important issues. The authors point out that using mode of action (MoA) as the basis for assessing cumulative risks is too restrictive, noting that the Organisation for Economic Co-operation and Development (OECD) guidance on the conduct and design of chronic toxicity and carcinogenicity suggests that regulators should only focus on groupings of individual chemicals that are known *a*) to act via a common sequence of key events and processes, *b*) to act on a common target or tissue, and *c*) to produce a common adverse outcome (e.g., cancer). In fact, the framework of the hallmarks of cancer framework makes it clear that cumulative risk assessment should anticipate synergies of chemicals acting *a*) via dissimilar sequences or processes, *b*) on different target/tissues, and *c*) even if they do not by themselves produce a common adverse outcome. As a result, the task force called for additional research on these issues, and they have raised concerns that cumulative risk assessment methods that are based on common mechanisms of toxicity or common modes of action may be underestimating cancer-related risks of the everyday exposures that the population faces.

The current workshop built upon the Halifax Project effort to establish principles and guidelines for the future testing of the low-dose mixture hypothesis of carcinogenesis by identifying gaps in knowledge, developing a unique strategy for assessing those gaps, and considering opportunities to integrate low-dose mixture concepts into risk-based decision-making.

## Identifying Information Gaps

A number of critical information gaps were identified and discussed that need to be addressed to better evaluate the low-dose mixture hypothesis of carcinogenesis and facilitate recommendations for future research regarding the impact of environmental factors on human health. The following topic areas outline the most critical information gaps.

### Understanding Carcinogenesis

The low-dose mixture hypothesis of carcinogenesis was established using the hallmarks of cancer outlined by Hanahan and Weinberg (2011) as an organizing framework. However, many of the teams lamented the fact that the processes of carcinogenesis are not well described in the experimental literature. In particular, we need to better understand the timing, sufficiency and relevance of individual hallmark events, and their combinations as they relate to the process of carcinogenesis (Schwarzman et al. 2015). Current information as to the process of cellular transformation has not been thoroughly reviewed, and there are also additional hallmarks of cancer and characteristics of carcinogens yet to be considered (Smith et al. 2016). Thus a thorough review of the current literature on all hallmarks of cancer and their interactions should open new avenues for future research that will help us evaluate the low-dose mixture hypothesis of carcinogenesis.

Additionally, there is a need for basic research using specific combinations of chemicals that affect individual and multiple hallmarks of cancer to provide a greater understanding of how the disease manifests. This hypothesis is predicated on the ideas that the multi-stage, multi-step progression of cancer may be influenced by pro-carcinogenic disruptions that are supportive of each of the hallmarks and facilitate cooperation from within the tumor microenvironment. A mutation-based risk assessment process misses the dynamics of epigenetic modulation during carcinogenesis, therefore, it is impossible to draw conclusions about the effects that low-dose exposures to mixtures of disruptive chemicals might produce. Tissue fate cannot be dictated uniquely by tumor cells. Interactions among these cells, the surrounding environment, and the host are keys to identifying the mechanisms of differentiation for aggressive (lethal) versus indolent (nonlethal) behaviors. For example, people who have been exposed to environmental mutagens (e.g., ultraviolet light, cigarette smoke) have a very high mutation rate in specific cancer types (e.g., skin and lung) (Srivastava et al. 2016). Therefore, research should focus on synergistic mixtures of disruptive chemicals that are known to affect specific tissues where aggressive cancers are more readily identified. We need a research agenda that includes a thorough investigation into the origins, determinants, and both biological (spatial) and epidemiological (temporal) evolution of the cancer hallmarks.

Future research will need to utilize tools that can investigate basic cellular- and tissue-level mechanisms to elucidate the various steps and pathways that are involved in the process. To that end, common environmental chemicals with known low-dose effects should be employed to simultaneously test their effects in sequence and in combination. In particular, it would be helpful to know how the chemical induction of certain combinations of hallmarks might advance or impede carcinogenesis, so chemical mixtures research that can illustrate these interactions should be aggressively pursued. Transgenic animal models of spontaneous carcinomas could be employed to determine the impact of low-dose mixtures of environmental chemicals on accelerating or impeding carcinogenesis.

Indeed, the human population is chronically exposed to low doses of many environmental chemicals. Using chemicals known to exert low-dose, hallmark-enabling effects, investigators can now selectively group and specifically design complex mixtures to produce pro-carcinogenic interactions in an effort to determine whether these mixtures actually produce predictable carcinogenic outcomes at environmentally relevant dose levels. This basic research is a critical step in testing the low-dose mixture hypothesis of carcinogenesis because it may reveal key hallmarks or combinations of hallmarks that can produce irreversible carcinogenic synergies. For example, the latency periods seen in carcinogenesis may be accelerated if we better understand how the sequential induction of these hallmarks allows the disease to progress and unfold.

### Biomarker Discovery

Cancer is a disease that involves changes in the health status of cells and tissues that ultimately lead to malignant tumors. It can originate at almost any location in the body. Generally, it is considered to involve an accumulation of multiple genetic, epigenetic, and physiological alterations that typically emerge over a long period of time. However, similar alterations can also be detected in response to injury, but injury-induced alterations rarely lead to malignant transformation. Therefore, a priority in the field of cancer research is to develop biomarkers that can distinguish genetic and epigenetic alterations that facilitate malignant transformation from those that are not relevant to cancer. Presence of common pathways, as well as the crosstalk between different pathways following exposure to carcinogens or toxic injury, adds to the complexity of the picture. This complexity calls for multidisciplinary research on biomarker discovery. Cancer biomarker research is a crowded field that requires innovative approaches in order to realize breakthroughs. Recent advances in molecular approaches using high-throughput molecular analysis technologies, including whole exome sequencing (WES) and microRNA profiling, offer hope for new discoveries in the field of cancer biomarker research (Srivastava et al. 2016). Development of transgenic mice models of early stages of carcinogenesis (McFadden et al. 2014), biobank repositories for clinical specimens, and associations seen in epidemiological studies are key resources for biomarker discovery research. Beyond what is currently known, these approaches and resources can be utilized to better identify signatures of cancer, or vulnerable states that may lead to cancer, in order to be able to evaluate predictable interactions that we might expect from cumulative exposures to (ostensibly noncarcinogenic) chemicals that act on key mechanisms and pathways.

### Dormancy, Latency, and Immune Evasion

Transformed cells will not necessarily establish cancer as long as cellular homeostasis keeps them in a dormant state. Dormant tumor cells have been detected in autopsies of individuals who were cancer free and died from noncancer causes (Folkman and Kalluri 2004). The presence of dormant malignant cells before a cancer is clinically apparent suggests that many cancer-free individuals may actually be cancer survivors because of harboring dormant malignant cells, which could establish a primary cancer at any time. Detection of such dormant cells requires advancements in our diagnostic technologies to investigate the impact of low-dose chemicals on the establishment of cancer dormancy before clinical cancer is evident, as well as on the shift to a nondormant state. This could in turn lead to advances in targeted therapies to prevent cancer development (Manjili 2014). However, the nature of tumor dormancy is not well characterized. We do not know whether tumor dormancy is a form of cellular transformation in stem cells or representative of a latency period during tumorigenesis. To this end, the role of immune surveillance and other key defense mechanisms (i.e., DNA repair, tumor suppressors, tumor stroma) in maintaining transformed cells in a dormant state early during chemical exposure are of primary interest. Similarly, the impact of chemicals on all of these defense mechanisms as it relates to the escape from dormancy and the establishment of primary cancers is fundamentally important and warrants careful investigation.

## Research Methodology

Previous chemical mixture studies have been conducted in rodents to evaluate effects on the incidence of cancer. Dose-additive carcinogenicity has been observed for a defined mixture affecting the same MoA, for example dioxin-like compounds (Walker et al. 2005). In addition, regulatory agencies have assessed combined effects of chemicals and chemical classes, including on cancer end points. However, the vast majority of mixture studies focus on structurally similar compounds acting through the same MoA. No one has systematically addressed all the hallmarks of cancer and assessed different combinations of structurally dissimilar agents, acting in concert to cause cancer, that individually are not known to be carcinogenic even at higher doses. A recent study on the characteristics of carcinogens has taken inspiration from chemicals known to cause cancer (Smith et al. 2016). This work should be extended and combined with in-depth study of cancer hallmark processes to facilitate application to chemicals with unknown risk, especially in combined exposure scenarios. The low-dose mixture hypothesis proposed in the Halifax Project highlights some of the limitations of existing regulation standards (Goodson et al. 2015). Current cancer risk assessment is based on biological responses, induced by the cumulative effects of exposure to individual carcinogens that act via a common sequence of key events and processes on a common target. Current research methodologies can be adapted to investigate the low-dose mixture hypothesis of carcinogenesis and identify circumstances under which we would expect chemical mixtures to act as carcinogens. However, this research has to consider the complexity of multi-step development patterns, and, in most cases, the existence of a long-latency period.

While addressing this question, it is important to consider the much-debated argument in the scientific community that animal-based models may be inadequate to predict human response to exposure. The National Research Council Report “Toxicity Testing in the 21st Century: a Vision and a Strategy (2007)” (NRC 2007) promoted the use of nonanimal models and called for embracing new technologies and basing assessments on toxicological mechanisms related to human biology and exposure. The proposed suite of tools includes the combination of computational models (integrative and targeted quantitative structure–activity predictions) and multiple *in vitro* assays, pathway-based approaches and toxicokinetic modeling (Browne et al. 2015; Bisson 2012; Zhao and Hartung 2015; Perkins et al. 2014; Kleinstreuer et al. 2014; Tice et al. 2013; Kavlock et al. 2012).

One important question is how do we measure success? A key point raised in the Halifax Project is that most of the hallmarks of cancer reflect properties of cells that are already transformed to be carcinogenic, so it is possible the hallmarks do not perfectly represent the key events or perturbations of normal cells (early in the onset of transformation) that precede or eventually lead to cancer. As such, further investigation is needed with regard to what happens in normal cells and tissues during cell latency from the time of exposure to cancer development. The Human Toxome Project, which focused on the concept of evidence-based medicine, was designed to develop the concepts and means for deducing, validating, and sharing molecular pathways of toxicity (PoT) (Bouhifd et al. 2015). Concurrently, the Adverse Outcome Pathway (AOP) project (Kleinstreuer et al. 2013; Langley et al. 2015), spearheaded by the OECD, strives to identify key molecular-, cellular-, and tissue-level events that lead to cancer and other toxicities. The goals of PoT and AOP are to build molecular annotations of network perturbations and then to establish causative correlations with biological phenotypic data. Higher confidence should be placed in the mechanistic understanding of pathways, rather than single end point approaches. By elucidating the underlying molecular mechanisms of carcinogenesis, investigators will be able to differentiate whether perturbations within a biological system are normal modulations within the homeostatic range or are early molecular-initiating events on the pathway to cancer.

Carcinogenesis is an adaptive process in a given tissue. Investigators need to look thoroughly at the biological events involved from both a spatial and temporal angle. If we utilize environmental mixtures as chemical probes to understand the biology, which methodologies do we identify as being helpful in this regard? One possibility is the use of an experimental toolbox that rapidly studies cancer development in real time in an entirely human tissue setting, complete with human epithelium and stroma from multiple tissues in correct three-dimensional (3-D) “seed and soil” architecture (Ridky et al. 2010). Although 3-D tissue cultures tend to be less sensitive than flat cell cultures for both the micronucleus assay and for measurements of cytotoxicity in air–liquid interface exposures, when combined within imaging and computational frameworks these models may provide a reliable method to identify key cellular pathways and elements in both tumor and stromal cells, necessary for oncogenesis (Casey et al. 2015).

Another research option is to characterize and monitor biological pathways in normal tissues or pre-malignant lesions at different time points using metabolomics or transcriptomics (Zhao and Hartung 2015). These approaches are useful when considering *in vivo* metabolism or dynamic changes in gene expression at the level of RNA. A core tool to help predicting adverse effects of chemicals at different doses and time is toxicokinetics (Chang et al. 2015). Inter-species variations in sensitivity and toxicokinetics call for robust models, mimicking the biology and physiology of the human body and the species from which one is extrapolating.

Molecular epidemiology can also be used since it focuses on the correlation between the contribution of genetic and environmental risk factors identified at a molecular level and the etiology, distribution, and prevention of cancer across populations (Tan et al. 2016). Historical data on exposures to chemical mixtures in combination with other variables (e.g., stress, racial disparity) should be reviewed within the context of individual chemicals and the enablement of individual hallmarks (i.e., to predict carcinogenic synergies and inform multivariate analysis). Additionally, *in silico* integrative analyses in combination with biological data generated in a lab may provide additional support using an alternative mechanistic approach. In order to validate these types of studies for risk assessment, it will be important to establish the correct initial controls, and to enhance communication and data sharing between the epidemiologists, oncologists, toxicologists and biologists.

Large clinical data sets have been derived from patients and provide a wealth of untapped information. Bioinformatics and high-performance computing are key for successful data analyses and data integration. Clinical data on transgenerational effects in early carcinogenesis (e.g., inflammation and immune-system evasion pathways) raised important questions on the role of prenatal (*in utero*) exposure (Thompson et al. 2015). Epigenetic control and reprogramming drive non-coding microRNA expression and controls cell and tissue adaptation in response to adverse environmental changes. Epigenetics provides another attractive platform to study environmental mixtures and their biological effects in human target tissues.

Another emerging area of study that will provide valuable insight into the carcinogenic potential of environmental exposures is examining the microbiome. Microbes and microbiota affect carcinogenesis in three ways: *a*) altering the balance of tumor cell proliferation and death; *b*) regulating immune system function; and *c*) influencing metabolism of host-produced factors, nutrients, and drugs. In the gut, the microbiota detoxify, or intoxicate, dietary components, reducing inflammation and balancing host cell growth and proliferation. The modulation of the human microbiome by exogenous chemicals and metabolites could become a unique biological sensor to detect early phases of cancer development. It will be extremely informative to monitor these endogenous effects during particular time-windows of susceptibility (e.g., *in utero*, early childhood, menopause) and in sensitive subpopulations (e.g., immunocompromised, elderly).

It is possible to conduct preliminary short-term studies in low-cost, rapid model systems utilizing small model organisms such as zebrafish (adequate to include multiple mechanisms and end points), with combined mixtures. Further efforts to prioritize mixtures of interest and identify potential synergies based on chemical *in vivo* responses will inform early testing strategies. We also foresee the possibility of utilizing patient-driven organoids and specimens of pre-cancer lesions (prior to cellular transformation) for the screening of prioritized environmental chemicals. However, to support and validate such a hypothesis, we should use existing methods (Jones et al. 2008) or develop new molecular tools that are able to identify the critical sequence or timing of key early biological events, preferably in human cells and cohorts (Campbell et al. 2016; Feinberg et al. 2016). Likewise, tumor promotion by a given compound could be triggered by initiating genotoxicity induced by a second dissimilar compound. Mapping the sequence of biological events, and understanding what combinations are both necessary and sufficient to cause cancer, will also improve chemical prioritization and profiling and, thus, our understanding of the risk assessment of cumulative exposure. This is a hypothesis-generating, rather than hypothesis-testing strategy.

A new initiative has begun to identify and measure the exposome, an emerging concept representing the accumulation of chemical exposures of a person through their lifetime. The Human Exposome Project (http://humanexposomeproject.com) focuses on the totality of human exposure and is intended to help researchers discern some of the contributing factors that are driving chronic diseases, like cancer. Interrelated projects are expected to involve extensive biomonitoring (e.g., blood, urine, and saliva sampling) and other techniques to assess relevant biomarkers. The current information provides a strong basis for research development, but to best utilize this information investigators need a better mechanistic understanding of the process of human carcinogenesis and better early mechanistic markers of cancer development. The fields of drug discovery and risk assessment need not be mutually exclusive. Environmental mixtures can be used to understand the complexity of cancer biology. Ideally, clinicians would be able to accurately predict whether a lesion in a normal tissue will become malignant or remain in a chronic, benign stage (e.g., polyps in the colon or fibrocystic change in the breast). Improved early cancer detection and prediction of lethal versus nonlethal cancer progression is the basis to develop effective preventive and therapeutic strategies.

## Applications of the Low-Dose Mixture Hypothesis

As noted, the historic focus of research and policy has largely been to identify single chemicals that act as complete carcinogens and to attempt to regulate these chemicals accordingly. Many chemicals are tested to assess their toxicity and carcinogenic potential—complete carcinogenic potential—but many more are not. The results of the Halifax Project (Goodson et al. 2015) suggest that exposure to chemicals that are seemingly noncarcinogenic individually may have combined effects that will result in cancer as a disease end point, and that even the most rigorous testing standards in place today might not be sufficient. Application of the theory could have far reaching effects across scientific policy, but it would require revising long-held paradigms and restructuring of existing regulatory frameworks. How do we leverage the new information provided by the Halifax Project and harness novel research strategies to inform protective and preventative action, knowing that hundreds of new chemicals are introduced into the human exposome every year?

Although the NTP and the IARC have periodically used mechanistic data to establish chemical classifications, cancer risk assessment and hazard identification still rely heavily on multi-year animal tests, such as the NTP rodent bioassay (Bucher 2002), where tumor formation based on histopathological examination is the observed adverse outcome. In contrast, the U.S. Environmental Protection Agency (EPA) guidelines require such animal data to classify a substance as anything other than “Group D-Not Classifiable as to Human Carcinogenicity” (U.S. EPA 2005). While these tests are extremely valuable, they are expensive and low throughput, and may not adequately represent human cancer susceptibility arising from chronic exposure to mixtures of chemicals in the environment. To support future toxicological and regulatory practice, adverse effects must be clearly defined to include more than just cancer as an apical end point. Critical system state changes that represent key steps toward cancer should also be considered. Furthermore, risk assessment methodologies must do more to incorporate timing of exposure, susceptible populations, multiple stressors and real-world mixtures that are environmentally relevant.

The hallmark phenotypes of cancer, while all important biological processes, are not necessarily created equal. Understanding how each hallmark contributes to cancer progression, both in a temporal and a quantitative sense, will facilitate defining which combinations of perturbations can be characterized as adverse outcomes in terms of irreversible vulnerabilities. To move toxicological outcomes to intermediate, measurable stages of carcinogenesis requires a systems biology approach. Research must be done using time series data to understand tipping points (i.e., exposures beyond which cells or systems can no longer recover normal function (Shah et al. 2016). Adverse outcome pathway (AOP) networks relating molecular signaling perturbations to hallmark phenotypes on a cellular and tissue level can be tied to tumorigenic outcomes in whole animal models (Kleinstreuer et al. 2013), and these types of analyses should be extended with data from studies on complex mixtures. Generating these data will further clarify the relative contributions of each hallmark, and substantiate the mechanistic end points that can be measured as markers of cancer progression. A recent IARC review concluded that human carcinogens frequently exhibit 1 or more of 10 key characteristic properties (e.g., altering DNA repair, causing oxidative stress, inducing chronic inflammation, altering cell death) (Smith et al. 2016). Chemicals with these characteristics, especially in combination, may result in different types of mechanistic end points representative of multiple hallmark processes being activated. Understanding these carcinogenic characteristics on a chemical and molecular level, and measuring mechanistic end points on a cellular and tissue level, will be paramount to filling data gaps and building AOP networks to identify critical system state changes that can be incorporated into studies for risk assessment.

Testing environmentally relevant mixtures faces a number of challenges including sample sourcing, characterization, and understanding of component contributions. Using defined mixtures, where the individual chemicals are selected based on hypothesized or observed carcinogenic characteristics and hallmark contributions, has the advantage of amplifying interpretability of the results, especially when combined with *in vitro* bioactivity data on a molecular and cellular level for both the mixture and its constituents. However, environmental samples more accurately represent the true human exposure, and approaches for non-targeted screening of house dust, drinking water, etc. are being examined by organizations such as the U.S. EPA and the multi-national network of reference laboratories, research centers, and related organizations for monitoring of emerging environmental substances (see NORMAN Network, www.norman-network.net/). Both methods of sample identification are essential, but to provide a tractable data set that will influence regulatory practices it will be necessary to take a hybrid approach and use biomonitoring data to inform controlled mixtures that are environmentally relevant. The Halifax Project has already identified more than 80 chemicals that may be affecting cancer hallmark signaling, and this list could be cross-referenced with exposure data to create a suite of mixtures for further testing. Important considerations will be chemical coverage of hallmark processes, potential interactions between hallmarks, the influence of genetic susceptibility on the relative importance of certain hallmarks, and relevance of mixture compositions.

## Discussion and Conclusions

Considering that the utility of such research will be to inform policy and decision-making, the challenge will be to integrate various branches of science and data streams to develop a well-rounded approach to explore the low-dose mixture theory of carcinogenesis. Elements of such an approach would include the following topics:

Clinical epidemiology must develop new, long-term perspectives that account for sex and reporting differences upon exposure to chemical mixtures over time. For example, a limited perspective of the last four decades allows the impression that increases in breast cancer reflect overidentification of clinically insignificant disease through overzealous screening, possibly combined with effects of alcohol use, weight gain, and sedentary lifestyle. A broader picture will note that this argument was invoked 30 years ago to explain the increase in breast cancer from 1950 to late 1970s. It is unlikely that behavior changes have caused, first, a 30% increase in breast cancer and then a subsequent 20% increase in the incidence of invasive breast cancer. Overdiagnosis is also an inadequate etiology since it does not explain why male breast cancer, for which there are no screening programs, has increased at the same relative rate.
Translational toxicology must extend beyond guideline studies to adequately test the low-dose mixture hypothesis of carcinogenesis and provide mechanistic support for epidemiologic observations in human cohorts. By utilizing human 3-D cellular and tissue models and patient-driven material and data, researchers can begin to assess whether carcinogenic transformations can occur in response to mixtures of low dose, otherwise safe, chemical exposures.
Biological significance needs to guide the interpretation of experimental results within the context of normal human physiology. Carcinogenesis is an adaptive process in a given tissue and thus needs to be considered biologically from both a spatial and temporal perspective to identify and diagnose lethal vs non-lethal events pre- and post-cell malignancy. Research processes must specifically allow for an evolution in our understanding of how chemicals might interact with normal human physiology. For example, it has been asserted that bisphenol A (BPA) is safe because it is rapidly metabolized and excreted by humans (Teeguarden et al. 2011). However, the pattern of a peak level with rapid decline may mimic hormone “spikes” that drive normal sexual development, such as thelarche, and could act like an estrogen spike in a pre-pubertal female who would not otherwise experience daytime estrogen spikes.
Understanding the biology of early stages of carcinogenesis, including DNA repair, tumor suppressor genes, circulating tumor cells, tumor microenvironment, tumor promoting and associated inflammation, and the immune system evasion, play an important role in preventing the immortalization of human cells. Therefore, an emphasis should be placed on our understanding of the biology of early stages of carcinogenesis and on how chemical disruptions that specifically disable these processes may impact cancer susceptibility and the incidence of the disease. This will help the development of effective strategies for prevention and early detection.
Epigenetic and nongenotoxic effects of environmental chemicals remain a poorly studied area within cancer research. Since initial excitement from the first sequencing of the human genome 15 years ago, we have realized that epigenetic control of expression of a normal gene can be as important as whether or not that gene is mutated. For example, methylation of promoter (control) regions for genes or histone deacetylation are mechanisms through which specific genes may be regulated. The current data is in its infancy and results to date do not create a coherent picture. However, it is known that environmental chemicals can affect methylation of regulatory DNA sequences, including promoter regions, and therefore the limited understanding of the effects of many chemicals on gene expression is a serious knowledge gap.
Chemical diversity in biological activity and performance must be considered in the preparation of chemical libraries for testing the low-dose mixture hypothesis of carcinogenesis using high-throughput screening approaches. Investigations of chemicals that induce hallmarks of cancer should be expanded to include a broad array of hallmark-relevant mechanisms and a combination of structurally dissimilar compounds.
Computational low-dose and mixture models of human exposure must be developed to support low-dose mixture research with carcinogenesis pathway end points. Although useful in many ways, animal tests have significant limitations when predicting human response. The plethora of chemicals already in the environment makes unexposed control groups almost impossible to identify. Computational modeling of molecular and metabolic interactions between human cells or tissues and mixtures of low-dose chemicals would provide direct human-relevant data. Dissimilar structures and functions of chemicals must be studied in combinations with simultaneous exposure. Synergistic interactions may affect individual cells in ways that are not predicted by current single chemical exposure data. This is the message of the Halifax Project: It is no longer sufficient to assess chemical safety using individual chemicals.
Emerging technologies for future cancer biology and toxicology research must be supported by state-of-the-art technology. Molecular, imaging, and computational approaches continue to evolve in support of 3-D tissue modeling, high-performance computing, *in vivo* high-resolution imaging, biomarker screening and platforms for microbiomics, metabolomics, and transcriptomics.

To inform and evolve cancer risk assessment for low-dose mixtures, it will be necessary to produce and make accessible a critical mass of data as a basis to move from a conceptual framework to testable hypotheses. Research support should come from central governments across the world to address truly global issues like cancer incidence, mortality, and public health concerns in a coordinated fashion. Further, it should also come from stakeholders outside federal governments (e.g., industry and NGOs). Current cancer research funding is largely focused on therapeutics, but increasing awareness of the importance of early detection and prevention research is evidenced in several research initiatives. Examples include the Interagency Breast Cancer and Environmental Research Coordinating Committee (IBCERCC 2013), the California Breast Cancer Research Program (http://www.cbcrp.org/), the White House Cancer Moonshot Task Force (https://www.whitehouse.gov/the-press-office/2016/01/28/memorandum-white-house-cancer-moonshot-task-force) and the recent annual plan and budget proposal by the National Cancer Institute (http://www.cancer.gov/about-nci/budget/plan). Under joint programs, grant-funded researchers are beginning to work across scientific disciplines with racially and ethnically diverse communities to expand the study of risk factors that lead to breast cancer, including environmental exposures and windows of susceptibility. Success in a few key areas that build off the work of the Halifax Project and others will stimulate increased investment and, in turn, produce findings to inform policy decision-making. The implications of low-dose mixture theory extend far beyond the world of cancer, and the cumulative effects of chemical exposures could play a role in many other pathologies, emphasizing the need for a system that is dynamic and able to incorporate new research and findings.
